# Synergistic effects of repetitive transcranial magnetic stimulation and a dance movement intervention on mild cognitive impairment: study protocol for a four-arm factorial randomized controlled trial

**DOI:** 10.3389/fphys.2026.1931453

**Published:** 2026-09-09

**Authors:** Xuefeng Zhao, Qing He, Jiawen Yin, Xiaoqi Xu, Qiaoli Gong, Xuehai Lv

**Affiliations:** 1 Department of Martial Arts and Dance, Shenyang Sport University, Shenyang, China; 2 Department of Rehabilitation Medicine, Handan Central Hospital, Handan, China; 3 Department of Neurology, Handan Central Hospital, Handan, China

**Keywords:** dance, mild cognitive impairment, randomized controlled trial, rTMS, study protocol

## Abstract

**Background:**

Mild Cognitive Impairment (MCI) represents a critical transitional stage between normal cognitive aging and dementia, characterized by a high conversion rate to Alzheimer’s disease (AD). Current pharmacological interventions provide limited symptomatic relief and do not alter disease trajectory. Consequently, there is an urgent need for effective, multimodal, non-pharmacological interventions. While repetitive Transcranial Magnetic Stimulation (rTMS) and Dance Movement Intervention (DMI) individually show promise in mitigating cognitive decline, their combined synergistic effects in older adults remain uninvestigated. This protocol outlines a randomized controlled trial to evaluate the clinical efficacy of combining rTMS with a culturally tailored DMI (Chinese square dancing) for older adults with MCI.

**Methods:**

This study is designed as a four-arm, single-blinded, parallel-group randomized controlled trial. We aim to recruit 160 community-dwelling older adults (aged 50–85 years) diagnosed with MCI. Participants will be randomly allocated (1:1:1:1) to one of four groups: Combined Intervention (active rTMS + DMI), rTMS Only (active rTMS + social control), DMI Only (sham rTMS + DMI), or a Control Group (sham rTMS + social control). The 12-week intervention phase utilizes intermittent theta burst stimulation (iTBS) targeted at the left dorsolateral prefrontal cortex (DLPFC) and a structured Chinese square dancing program. Assessments occur at baseline, 12 weeks (post-intervention), and 24 weeks (follow-up). The primary outcome is global cognitive function, measured by the Montreal Cognitive Assessment (MoCA). Secondary outcomes assess memory, executive function, electroencephalography (EEG) biomarkers of neuroplasticity (P300), physical performance, and quality of life.

**Discussion:**

This factorial trial will comprehensively evaluate the individual and interactive effects of neuromodulation and physical-cognitive training. By hypothesizing that rTMS will effectively prime cortical excitability to maximize the benefits of the complex sensorimotor demands of DMI, this study aims to establish a scalable, evidence-based multimodal therapy to delay cognitive decline in aging populations.

## 1 Introduction

Mild Cognitive Impairment (MCI) represents a transitional stage between normal aging and dementia, characterized by a decline in cognitive function greater than expected for an individual’s age and education level, but without significant interference with daily activities (Gauthier et al., 2006). The global prevalence of MCI is substantial and increasing, particularly among older adults, with estimates ranging from 10% to 20% in individuals over 65 years of age (Petersen, 2016). A significant concern is the high conversion rate of MCI to Alzheimer’s Disease (AD), with approximately 10–15% of individuals with MCI progressing to AD annually, compared to 1–2% in the general older population (Chen et al., 2022). This progression imposes a substantial global burden, including escalating healthcare costs, caregiver strain, and diminished quality of life for affected individuals and their families (Wimo et al., 2017). Effective interventions to prevent or delay conversion from MCI to AD are therefore a public health priority, making MCI a critical target for early intervention.

Despite extensive therapeutic strategies, current interventions for MCI have shown limited efficacy in altering disease progression or providing sustained cognitive benefits. Pharmacological treatments—primarily acetylcholinesterase inhibitors and NMDA receptor antagonists—offer modest symptomatic relief but do not halt neurodegeneration (Kavirajan and Schneider, 2007) and are often limited by side effects and poor adherence. Non-pharmacological approaches, including cognitive training, physical exercise, and dietary interventions, have demonstrated some promise in improving cognitive function and delaying decline (Livingston et al., 2020); however, their effects are often modest, and challenges remain regarding long-term adherence, scalability, and the precise mechanisms underlying their benefits. The heterogeneous nature of MCI also suggests that a single intervention may not be sufficient to address the multifaceted pathology and diverse clinical presentations. Consequently, there is a critical need for novel multi-modal interventions that can synergistically target the complex neurobiological and behavioral aspects of MCI.

Repetitive Transcranial Magnetic Stimulation (rTMS) has emerged as a non-invasive brain stimulation technique with significant therapeutic potential for neurological and psychiatric disorders, including MCI. rTMS operates by generating a rapidly varying magnetic field over the scalp, inducing a transitory electric current in the cortical surface. This current modulates neuronal activity directly beneath the coil and in connected brain regions, enhancing cortical excitability and promoting long-term potentiation (LTP)-like plasticity (Esposito et al., 2022). Specifically, facilitatory protocols such as intermittent theta burst stimulation (iTBS) deliver bursts of high-frequency stimulation (50 Hz) repeated at a theta rhythm (5 Hz), mimicking endogenous learning rhythms in the brain (Sharbafshaaer et al., 2024).

Previous studies have demonstrated that rTMS, particularly when applied to the dorsolateral prefrontal cortex (DLPFC), a region critical for executive function and memory, can improve cognitive performance in individuals with MCI (Guo et al., 2025; Yuan et al., 2025). Recent comparative trials indicate that iTBS over the left DLPFC yields cognitive benefits equivalent to conventional high-frequency rTMS in MCI, while requiring only a fraction of the session time (e.g., 3 vs. 37.5 minutes) (Zheng et al., 2024). rTMS can also modulate functional connectivity within key brain networks, such as the default mode network (DMN) and fronto-parietal network, which are often disrupted in MCI (Esposito et al., 2022; Guo et al., 2025). By directly influencing neural circuits and promoting synaptic plasticity, rTMS may prime the brain to be more receptive to subsequent cognitive and behavioral interventions in MCI. Among various forms of physical activity, dance stands out as a particularly rich, multi-faceted intervention for older adults with MCI. Dance—particularly structured forms such as Chinese square dancing—integrates physical, cognitive, social, and emotional components, offering benefits beyond conventional aerobic exercise (Colcombe and Kramer, 2003; Tao et al., 2023; Wu et al., 2022). It improves cardiovascular fitness, balance, gait speed, and motor coordination, all vital for maintaining physical independence and reducing fall risk in MCI (Sánchez-Alcalá et al., 2025). Learning and executing complex dance sequences demand continuous attention, working memory, planning, and decision-making, providing intensive cognitive training, while synchronizing movements to music and partners further challenges dual-tasking abilities and cognitive flexibility (Quan et al., 2025). Group dance activities foster social engagement and a sense of community—reducing loneliness and isolation, which are known risk factors for cognitive decline and depression in MCI (Cacioppo and Cacioppo, 2018)—while the accompanying music, rhythmic movements, and expressive elements can enhance mood, reduce stress, and provide multisensory input that supports brain health and emotional regulation (Hewston et al., 2021). Chinese square dancing, with its energetic rhythms and group format, is particularly well-suited to delivering these benefits, as it is easy to learn, affordable, and highly popular among older adults in China (Ou et al., 2022).

While both rTMS and dance interventions individually show promise in ameliorating cognitive decline in MCI, their combination is hypothesized to yield synergistic effects that surpass the benefits of either intervention alone. The rationale is grounded in the distinct yet complementary neurobiological and behavioral mechanisms of each modality. Critically, rTMS and dance converge on a shared set of physiological mechanisms—BDNF signaling, cortical excitability, motor-cognitive integration, and neuroplasticity—providing a unifying biological substrate for their synergy. rTMS can induce a state of enhanced cortical excitability and synaptic plasticity, particularly in the DLPFC and its interconnected networks, such as the hippocampus (Esposito et al., 2022; Phipps et al., 2021). This neuromodulatory effect can be conceptualized as ‘priming’ the brain, making it more receptive to the plastic changes induced by subsequent cognitive and physical training (Guo et al., 2025). Dance, with its rich sensorimotor and cognitive demands, can then capitalize on this primed state. Physical activity, including dance, is known to promote the release of neurotrophic factors such as Brain-Derived Neurotrophic Factor (BDNF) and Insulin-like Growth Factor 1 (IGF-1), enhance cerebral blood flow, and stimulate neurogenesis (Ren et al., 2025; J. Zhang et al., 2025). These activity-dependent neurobiological changes can consolidate and extend the neuroplastic effects initiated by rTMS, leading to more robust and sustained cognitive benefits in MCI (Yuan et al., 2025).

The cognitive demands inherent in learning and performing dance routines, such as memorizing steps, coordinating movements, and adapting to music, provide an ideal environment for cognitive rehabilitation. Combined with the targeted neural modulation from rTMS, which can enhance neural efficiency and connectivity in critical cognitive regions, this may yield more profound and lasting improvements in specific cognitive domains (e.g., executive function, memory, attention) and overall global cognition in MCI patients (Quan et al., 2025; Yuan et al., 2025). The engaging and enjoyable nature of dance may further improve adherence to rTMS sessions, maximizing the therapeutic benefits of both interventions. The combined intervention offers a comprehensive approach by simultaneously targeting the neurological underpinnings of MCI through rTMS and addressing the physical, cognitive, social, and emotional dimensions through dance—a multi-modal framework essential for managing a condition as complex as MCI, which affects multiple aspects of an individual’s well-being.

**Figure 1:**
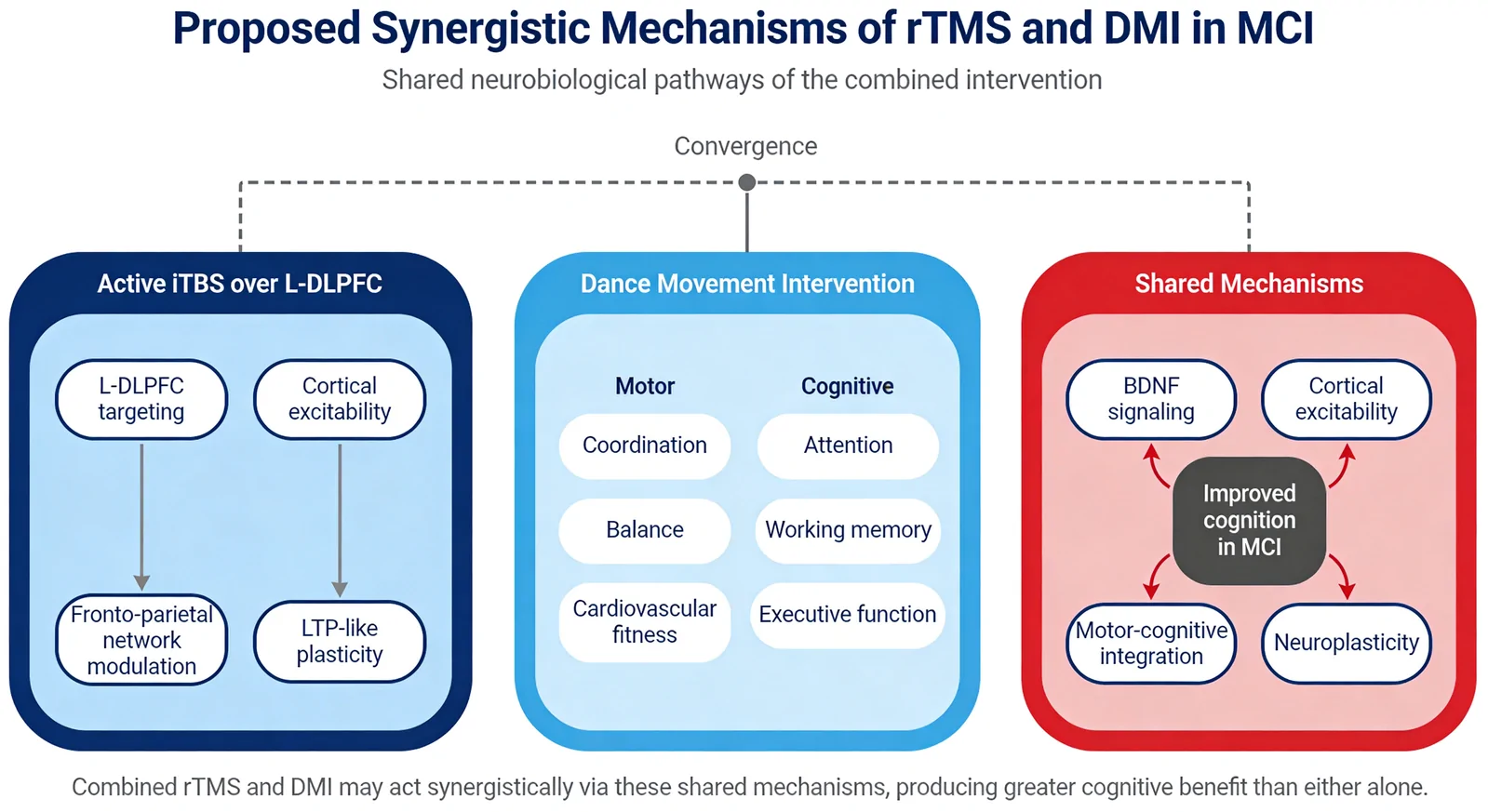
Proposed synergistic mechanisms of rTMS and DMI in MCI. The figure illustrates the biological rationale underlying the combination of repetitive transcranial magnetic stimulation (rTMS) and Dance Movement Intervention (DMI) in older adults with mild cognitive impairment (MCI). Left panel: rTMS is delivered as intermittent theta-burst stimulation (iTBS) over the left dorsolateral prefrontal cortex (L-DLPFC), which modulates cortical excitability, fronto-parietal network activity, and long-term potentiation (LTP)–like plasticity. Middle panel: DMI integrates motor components (coordination, balance, and cardiovascular fitness) with cognitive components (attention, working memory, motor planning, and executive function). Right panel: Both interventions converge on four shared neurobiological pathways—brain-derived neurotrophic factor (BDNF) signaling, cortical excitability, motor-cognitive integration, and neuroplasticity—which together are hypothesized to yield greater cognitive improvement in MCI than either modality alone.

Despite the compelling theoretical rationale for combining rTMS and dance, randomized controlled trials investigating their synergistic effects in older adults with MCI remain scarce. Existing research has largely focused on the individual effects of rTMS or various forms of exercise, including dance, but rarely on their combined impact using a robust factorial design. To our knowledge, no randomized controlled double-blind study has compared rTMS combined with Chinese square dance against appropriate controls, nor comprehensively disentangled the individual contributions of each modality. Therefore, the primary aim of this study is to evaluate the clinical efficacy of combined rTMS and Chinese square dance on cognitive function, neuroplasticity, and physical performance in older adults with MCI. Secondary aims include evaluating the individual effects of rTMS and Chinese square dance and elucidating the potential synergistic mechanisms underlying the combined intervention, thereby providing a robust rationale for future therapeutic strategies in MCI. **Figure 1** illustrates the proposed biological framework of this combined intervention, summarizing the four shared neurobiological pathways—BDNF signaling, cortical excitability, motor-cognitive integration, and neuroplasticity—on which rTMS and DMI are hypothesized to converge to enhance cognitive outcomes in MCI.

## 2 Methods and analysis

### 2.1 Trial design

**Figure 2:**
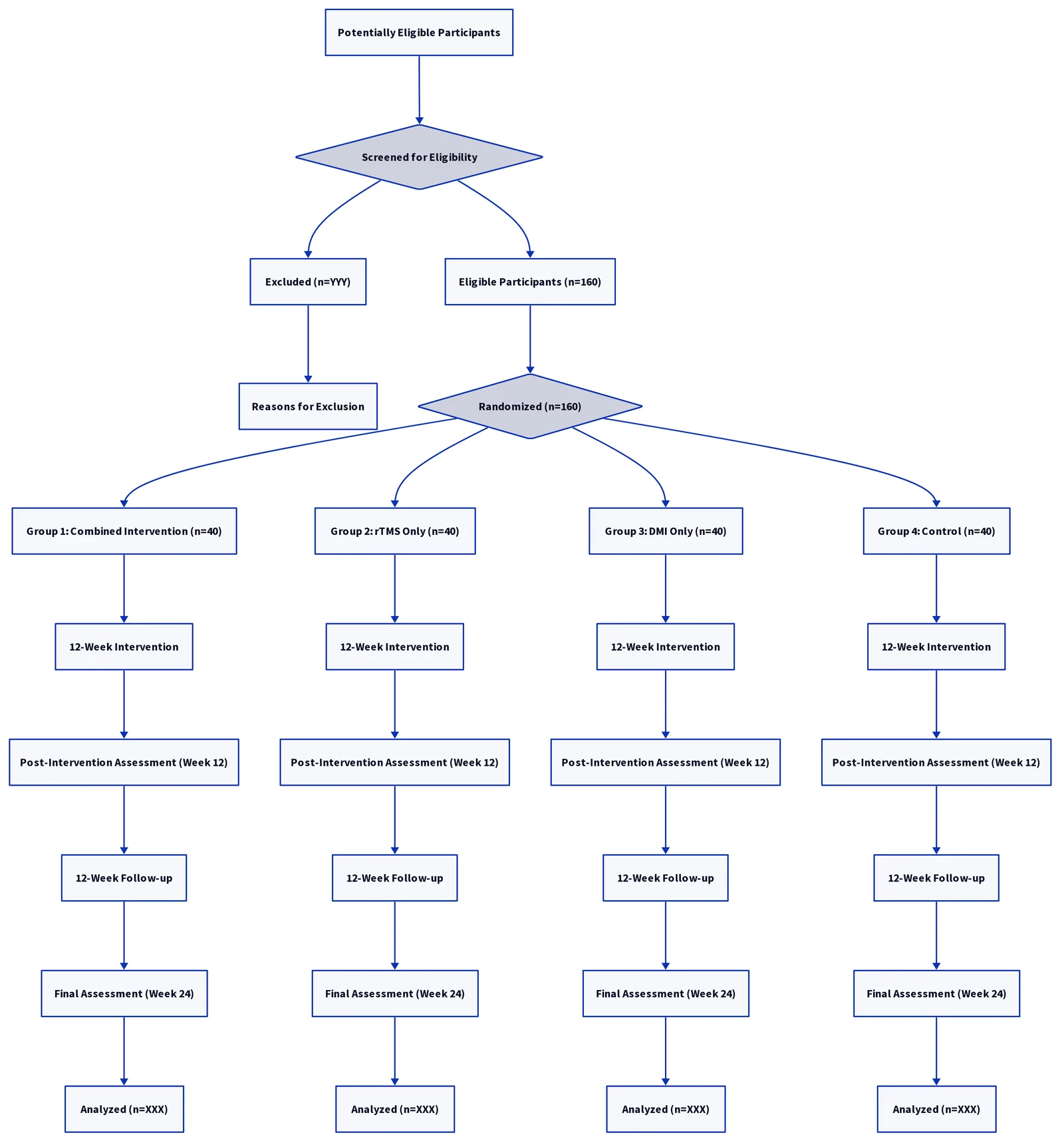
CONSORT flow diagram illustrating the participant screening, randomization, and allocation process for the four-arm factorial randomized controlled trial. Potentially eligible participants are screened for eligibility and, following exclusion of ineligible individuals, 160 participants are randomized (1:1:1:1) into four parallel groups: Group 1, Combined Intervention (active rTMS + DMI, n = 40); Group 2, rTMS Only (active rTMS + social control, n = 40); Group 3, DMI Only (sham rTMS + DMI, n = 40); and Group 4, Control (sham rTMS + social control, n = 40). Each group undergoes a 12-week intervention phase, a post-intervention assessment at Week 12, a 12-week follow-up phase, and a final assessment at Week 24. rTMS, repetitive transcranial magnetic stimulation; DMI, dance movement intervention.

This study will adopt a four-arm, single-blinded, parallel-group randomized controlled trial (RCT) design, chosen to comprehensively evaluate the individual and synergistic effects of repetitive transcranial magnetic stimulation (rTMS) and a dance movement intervention (DMI). Participants will be randomly assigned in a 1:1:1:1 ratio to one of four parallel groups: (1) a Combined Intervention Group, receiving active rTMS followed by the DMI (Chinese Square Dance); (2) an rTMS Only Group, receiving active rTMS and a time-matched, non-aerobic social activity control; (3) a DMI Only Group, receiving sham rTMS followed by the DMI; and (4) a Control Group, receiving sham rTMS and the social activity control. The research protocol will adhere to the Standard Protocol Items: Recommendations for Interventional Trials (SPIRIT) guidelines (Chan et al., 2013) and the Consolidated Standards of Reporting Trials (CONSORT) statement for non-pharmacologic treatments (Barbour et al., 2017), and the study will be conducted in accordance with the Declaration of Helsinki (Association, 2025) and has been registered at Chinese Clinical Trial Registry (ChiCTR2600127655). All procedures will be carried out at the Department of Rehabilitation, Handan Central Hospital, Handan, China (see **Figure 2**).

### 2.2 Participants

Participants will be recruited from the Department of Rehabilitation at Handan Central Hospital. A convenience sampling method will be employed, with local physicians providing a list of potentially eligible patients diagnosed with MCI. All participants will provide written informed consent before enrollment. A family member or legal guardian will also be asked to co-sign the consent form. Participant data will be anonymized to ensure confidentiality. The data will be secured during the study and destroyed five years after the study completion.

Inclusion criteria: (1) Diagnosis of amnestic or multi-domain MCI by a specialist physician according to the National Institute on Aging-Alzheimer’s Association (NIA-AA) criteria. (2) Age between 50 and 85 years. (3) Montreal Cognitive Assessment (MoCA) score between 18 and 25. (4) Geriatric Depression Scale (GDS-15) score < 5. (5) Physically able to participate in dance activities without assistive devices and deemed at low risk for falls (Berg Balance Scale score ≥ 41). (6) Stable doses of any prescribed medications for at least 30 days prior to screening. (7) Sufficient visual and auditory acuity (with correction if necessary) to complete assessments and interventions. (8) Voluntarily agrees to participate and provides informed consent.

Exclusion criteria: (1) Diagnosis of dementia (any type). (2) Presence of major psychiatric conditions (e.g., major depressive disorder, schizophrenia) or neurological disorders (e.g., Parkinson’s disease, stroke) that could confound cognitive assessment. (3) Contraindications to rTMS (e.g., history of seizures, metallic implants in the head). (4) Regular participation in structured exercise (≥ 3 times per week) or another cognitive intervention program within the past six months. (5) Abuse of drugs or alcohol that could cause cognitive impairment.

The study would cease if any of the following occurs: (1) Participant’s death. (2) Medical problems related to exercises (fracture, cardiac or cardiopulmonary problems, etc.). (3) Participants experience adverse event(s) that require discontinuation in the judgment of the principal investigator or trial instructor. (4) Participants have a need for additional intervention (such as cognitive treatment) that would interfere with the trial. (5) Participant neglects to follow trial instructions. (6) Participant have lost more than six sessions and cannot make additional lessons to cover the deficiency. (7) Participants or their legally authorized representative request consent withdrawal. An adverse event will be defined as any untoward medical occurrence in a participant during participation in the study. Adverse events include unbearable discomfort with rTMS intervention, falling, tripping, serious muscle soreness during dance participation. Participants will be allowed to finish all dance classes in a sitting position to prevent further adverse events if necessary.

### 2.3 Intervention protocol

The study consists of a 12-week intensive intervention phase followed by a 12-week follow-up phase. This 12-week duration is chosen based on evidence that significant cognitive and neuroplastic changes in MCI populations typically manifest within 3 months of rTMS and structured exercise (e.g., dance) (Guo et al., 2025; Sánchez-Alcalá et al., 2025). While a 24-week intervention was considered, the 12-week model is preferred to maximize participant retention and minimize “intervention fatigue” in this elderly population, while still being sufficient to demonstrate synergistic effects (Ren et al., 2025). The subsequent 12-week follow-up will allow for the assessment of the durability of these effects—a critical factor for clinical translation. Participants will be supervised by trained instructors and medical staff.

#### 2.3.1 rTMS intervention (active and sham)

Participants in the Combined Intervention and rTMS Only groups will receive active rTMS. Sessions will be conducted 5 days per week for the first 4 weeks (induction phase), followed by 2 sessions per week for the remaining 8 weeks (maintenance phase). Each session will involve intermittent theta burst stimulation (iTBS) over the left dorsolateral prefrontal cortex (DLPFC). The iTBS protocol consists of bursts of 3 pulses at 50 Hz, repeated at 5 Hz. A total of 600 pulses will be delivered per session (2 seconds on, 8 seconds off, for 190 seconds) at 70–80% of the individual’s resting motor threshold (rMT). A 7-cm figure-of-eight coil connected to a magnetic simulator will be used, with neuronavigation to ensure consistent targeting.

For participants in the Combined Intervention group, the sequence and timing of the two modalities are strictly controlled to maximize the neuroplastic priming effect. The iTBS will always be administered prior to the DMI. Following the completion of the 3-minute iTBS session, participants will commence the DMI within a strict 10- to 15-minute window. This specific timing interval is chosen based on neurophysiological evidence demonstrating that a single block of iTBS induces a transient state of enhanced cortical excitability and long-term potentiation (LTP)-like plasticity that peaks shortly after stimulation and lasts for approximately 20 to 30 minutes (Huang et al., 2005; Tse et al., 2018). Initiating the cognitively and physically demanding dance routines within this critical time window ensures that the activity-dependent neuroplasticity driven by the DMI coincides with the iTBS-induced state of heightened cortical receptivity, thereby optimizing the synergistic therapeutic effects (Stoykov and Madhavan, 2015).

Participants in the DMI Only and Control groups will undergo a sham rTMS procedure. A placebo coil that produces an equivalent sound and scalp sensation without inducing a therapeutic magnetic field will be used to maintain blinding.

#### 2.3.2 Dance movement intervention

Participants in the Combined Intervention and DMI Only groups will participate in a structured Chinese Square Dancing program. Sessions will be held 3 times per week for 12 weeks, with each session lasting 60 minutes. The intervention emphasizes rhythm, coordination, memory of sequences, and social interaction. Participants in this group will receive in total thirty-six hours of intervention. A maximum of 10 participants will be enrolled in each DMI class to ensure performance quality. Instructors will use oral explanations and encouragements during the interventions.

Chinese square dancing movement programs have been applied within various populations showing evidence of benefits (Bisbe et al., 2020; Blumen et al., 2022; Zhu et al., 2018, 2022). Any 4 × 4 beats music can be used for dance intervention routine; participants will also be allowed to recommend music. The DMI will combine choreography and improvisation, single and partner strategies, including gentle body movements (consisting of limbs and spine movements) with special emphasis on rhythm and contralateral movement on both sides of the body to enhance coordination and stimulation between right and left cerebral hemispheres (Ho et al., 2015, 2020). The DMI routine contains repetition and improvisation; therefore, it will require memory, concentration and dual-task function to complete the dance correctly.

The dance moves are gender-neutral and applicable to both males and females. Dance routines will be adapted to participants’ abilities, and new routines may be introduced every three to four weeks. Each session will be led by one dance instructor positioned at the front of the group, who will lead the dance and encourage social connection. A second instructor and supporting medical staff will supervise participants’ movements, guide correct execution, and ensure safety. The DMI targets both motor participation and specific cognitive processes. At the motor level, the choreographed and improvised routines are designed to engage whole-body coordination, balance, and cardiovascular demand. At the cognitive level, the routines are designed to engage attention, working memory, motor planning, and executive function—through memorizing step sequences, synchronizing movements to music, adapting to changing choreographic patterns, and engaging in partner-based interactions. Participants are not required to perform movements with 100% accuracy; rather, the emphasis is on sustained engagement with both the motor and cognitive demands of the task, in order to experience the body as a source of creativity and reliability. A practice video featuring standard movements will be provided after each class to support at-home familiarization. Rest periods, during which participants may sit and drink water, are built into each session. To accommodate older participants, the dance intervention can be performed in seated positions when needed to reduce fatigue. Intervention fidelity and adequate involvement will be documented session-by-session using a structured adherence log completed by the supervising instructor. The log will capture: (i) session attendance and duration of active participation; (ii) instructor-rated movement engagement and quality on a 5-point Likert scale; (iii) proportion of the session completed in standing versus seated position; and (iv) any adverse events or safety concerns. These data will be used to characterize intervention delivery and to support per-protocol analyses.

Each dance class will consist of six structured components, delivered in the following sequence:

1. Warm-up (10 min) — stepping combined with breathing, head movement, spinal side-bending, and turning exercises.
2. DMI choreography (30 min) — three structured routines performed in unison to music; the third routine includes interactive movements with partners.
3. Break (5 min) — seated rest and water intake.
4. DMI improvisation (15 min) — the instructor performs movements at the front of the group; participants may follow along, dance independently, or engage in interactive movements with partners.
5. Cool-down (5 min) — slow stretching and breathing, combined with low-speed stepping.
6. Social sharing (10 min) — facilitated group discussion in which each participant is invited to use one or two words, a gesture, or a movement to express their feelings, enjoyment, and playfulness regarding the dance experience, and to share words of encouragement or care with one another.

These six steps aim to encourage the participants to remember steps and sequences; concentration and memory will be required to perform this dance routine smoothly and correctly; improve mood and vitality, foster imagination and creativity. In addition, attention and planning movement in space with the aim of using movement activities and paying attention to bodily experiences to improve cognitive function. Furthermore, personal expression (verbal and non-verbal) to enhance communication and social exchange, respectively. For treatment fidelity, before the intervention, two instructors will teach two classes as practice for dance intervention groups. Every class will last one hour, with a one-day gap between the two classes. In these sessions, researchers will explain the essentials of DMI—including the six-component class structure (warm-up, choreography, break, improvisation, cool-down, and social sharing), safety guidelines (footwear, hydration, permission to rest in a seated position at any time, and the principle that movements need not be 100% accurate), and use of the post-class practice video—together with its potential health benefits for older adults with MCI, including improvements in motor function (balance, coordination, cardiovascular fitness), cognitive function (attention, working memory, motor planning, executive function), social engagement, and emotional well-being. Participants will then be introduced to basic movements through guided practice with instructor feedback.

A sign-in system will be implemented for each class during the investigation, and a small gift or daily necessities (towel, soap, toothbrush, etc.) will be awarded to those participants who attended intervention sessions. The aim of training monitoring is to check exercise interventions’ training intensity. The interventions will be conducted as moderate intensity, based on levels of perceived exertion. Participants will be instructed to carry out their intervention activities without exceeding 4–6 points on the Borg Scale (0 to 10 scale) (Borg, 1982). Instructors will regularly ask participants about their perceived fatigue levels during activities and help them to maintain the target level of effort.

#### 2.3.3 Social activity control

To control for the social interaction and general engagement inherent in the DMI, participants in the rTMS Only and Control groups will attend time-matched sessions (60 minutes, 3 times per week) involving non-aerobic activities such as watching educational videos, listening to music, and engaging in supervised, non-strenuous group discussions.

### 2.4 Measurement outcomes

Assessments will be conducted at three time points: Baseline (T0), Post-Intervention (T1, Week 12), and Follow-up (T2, Week 24). T1 assessments will be conducted 24–72 hours after the final intervention session of Week 12, to avoid the potential confounding influence of acute rTMS and dance exposure on cognitive and physical performance. To control for circadian variation, T1 and T2 assessments for each participant will be scheduled at the same time of day as that participant’s T0 assessment. Participants’ self-rated scales will be completed under guidance. All the tests will be administered in accordance with the standard procedures. The specific schedule of enrolment, interventions, and assessments is detailed in **Table 1**.

**Table 1:** SPIRIT schedule of enrolment, interventions, and assessments.

Procedure	*Enrolment*	*Allocation*	*Intervention Phase*	*Post-Intervention*	*Follow-up*
*Timepoint*	-t_1_ (Screening)	T0 (Baseline)	Weeks 1–12	T1 (Week 12)	T2 (Week 24)
Enrolment:					
*Eligibility Screening*	**X**				
*Informed Consent*	**X**				
*Demographics & Medical History*	**X**				
*Inclusion MoCA, GDS-15, BBS*	**X**				
*Randomization*		**X**			
Interventions:					
*Active or Sham iTBS (rTMS)*			**X**		
*Chinese Square Dancing (DMI)*			**X**		
*Social Activity Control*			**X**		
Assessments:					
Primary outcome					
*Montreal Cognitive Assessment (MoCA)*		**X**		**X**	**X**
Secondary outcomes					
*Wechsler Memory Scale (WMS-IV)*		**X**		**X**	**X**
*Trail Making Test (Part A & B)*		**X**		**X**	**X**
*Berg Balance Scale (BBS)*		**X**		**X**	**X**
*6-Minute Walk Test (6MWT)*		**X**		**X**	**X**
*12-item Short Form Survey (SF-12)*		**X**		**X**	**X**
Neurophysiological & exploratory					
*EEG/ERP (P300 Oddball paradigm)*		**X**		**X**	
*Resting-State fMRI (rs-fMRI)*		**X**		**X**	
Safety & fidelity					
*Adverse Events Monitoring*			**X**	**X**	
*Blinding Integrity Questionnaire*				**X**	
*Qualitative Interviews*					**X**

-t₁: Initial screening phase to determine clinical eligibility based on MoCA, Geriatric Depression Scale (GDS-15), and Berg Balance Scale (BBS) inclusion criteria. T0: Baseline assessment conducted immediately prior to randomization. T1: Primary endpoint assessment conducted immediately following the 12-week intervention. T2: Follow-up assessment conducted 12 weeks post-intervention to determine durability of effects. MoCA, Montreal Cognitive Assessment; GDS-15, 15-item Geriatric Depression Scale; WMS-IV, Wechsler Memory Scale Fourth Edition; BBS, Berg Balance Scale; 6MWT, 6-Minute Walk Test; SF-12, 12-item Short Form Health Survey; EEG/ERP, Electroencephalography/Event-Related Potential; rs-fMRI, Resting-state Functional Magnetic Resonance Imaging.

#### 2.4.1 Primary outcome

The primary outcome measure is the change in global cognitive function from baseline (T0) to post-intervention (T1), assessed using the Montreal Cognitive Assessment (MoCA). The MoCA is a highly sensitive, 30-point screening tool specifically designed to detect mild cognitive impairment (MCI), assessing multiple cognitive domains including executive function, visuospatial ability, memory, attention, and language (Nasreddine et al., 2005). To determine the clinical relevance of any observed changes, we will apply a Minimal Clinically Important Difference (MCID) threshold. Based on recent clinimetric evaluations in older adults with cognitive impairment, an increase of ≥ 2 points on the MoCA will be considered a clinically meaningful improvement (Lindvall et al., 2024).

While the MoCA is less susceptible to ceiling effects than the Mini-Mental State Examination (MMSE) in MCI populations, some participants with very mild impairment (e.g., baseline scores of 24–25) may still approach the maximum score of 30, potentially masking further cognitive gains (Trzepacz et al., 2015). To mitigate this, our statistical analysis plan will incorporate Tobit regression models, which are specifically designed to handle censored data and adjust for potential ceiling effects, ensuring accurate estimation of the intervention’s true cognitive impact (Proust-Lima et al., 2007).

#### 2.4.2 Secondary outcomes

Secondary outcomes encompass specific cognitive domains, physical performance, and quality of life, all measured at T0, T1, and T2. Memory will be assessed using the Wechsler Memory Scale, Fourth Edition (WMS-IV), while executive function will be evaluated via the Trail Making Test (Parts A and B). Physical performance will be measured using the 6-Minute Walk Test (6MWT) to assess functional exercise capacity and the Berg Balance Scale (BBS) to evaluate static and dynamic balance. Finally, quality of life will be assessed using the 12-item Short Form Health Survey (SF-12).

#### 2.4.3 Neurophysiological biomarkers

To objectively quantify changes in neuroplasticity and cognitive processing speed, the P300 event-related potential (ERP) will be recorded using electroencephalography (EEG) at baseline (T0) and post-intervention (T1). The P300 component will be elicited using a standardized two-stimulus auditory oddball paradigm, in which participants are instructed to press a button in response to an infrequent target tone (e.g., 2000 Hz, 20% probability) embedded within a series of frequent standard tones (e.g., 1000 Hz, 80% probability) (Costanzo et al., 2024).

The P300 is a robust, non-invasive biomarker of attention and working memory updating. In individuals with MCI, the P300 typically exhibits reduced peak amplitude and prolonged latency compared to healthy age-matched controls, reflecting slowed neural processing and reduced allocation of attentional resources (Demirayak et al., 2023). We will specifically analyze the P300 peak latency (in milliseconds) and peak amplitude (in microvolts) at midline electrodes (Fz, Cz, Pz), where the signal is most prominent. A reduction in P300 latency and an increase in amplitude post-intervention will serve as direct neurophysiological evidence of enhanced cortical efficiency and target engagement by the iTBS and dance interventions (Stuckenschneider et al., 2020).

#### 2.4.4 Exploratory outcome measure

Resting-state functional connectivity (rs-fMRI). To investigate the underlying neuroplastic mechanisms of the interventions, resting-state functional magnetic resonance imaging (rs-fMRI) will be acquired at T0 and T1. Analysis will focus on changes in functional connectivity within the Default Mode Network (DMN) and frontoparietal networks. rs-fMRI provides a task-free, low-burden method to quantify network-level neuroplasticity induced by iTBS and dance interventions in older adults with MCI (Balazova et al., 2021; H. Zhang et al., 2019).

### 2.5 Qualitative interviews (acceptability and feasibility)

To comprehensively understand the intervention’s acceptability, feasibility, and perceived impact, a qualitative component will be embedded within the randomized controlled trial. This mixed-methods approach will provide rich, in-depth insights that complement the quantitative outcome measures.

#### 2.5.1 Participants and sampling

A purposive sample of approximately 20–25 participants (5–7 from each intervention group, including the control group) will be recruited for individual semi-structured interviews. This sampling strategy will ensure representation across intervention arms and varying levels of engagement and outcomes. Additionally, 5–7 medical staff (e.g., neurologists, nurses, rTMS technicians, dance instructors) involved in the study will be interviewed to gather perspectives on implementation feasibility, challenges, and perceived benefits.

#### 2.5.2 Data collection

Individual semi-structured interviews will be conducted by a trained qualitative researcher (not involved in direct participant care or intervention delivery) at the end of the 12-week follow-up period (Week 24). Each interview is expected to last approximately 45–60 minutes. Interviews will be audio-recorded with explicit consent and transcribed verbatim. The interview guides will be developed based on relevant literature and the study objectives. For Participants: Acceptability of the intervention (e.g., comfort, enjoyment, perceived burden), satisfaction with the program, perceived cognitive and physical changes, social engagement, and suggestions for improvement. For Medical Staff: Feasibility of implementing the rTMS and dance interventions (e.g., logistical challenges, resource requirements, training needs), perceived participant engagement and response, and overall impressions of the program’s integration into clinical practice.

#### 2.5.3 Reporting

The qualitative findings will be reported in accordance with the Consolidated Criteria for Reporting Qualitative Research (COREQ) 32-item checklist (Tong et al., 2007).

### 2.6 Measures to ensure reliability and validity

To ensure the highest level of scientific rigor, this study incorporates robust measures to protect both the internal and external validity of the quantitative data, as well as the trustworthiness of the qualitative findings.

#### 2.6.1 Quantitative reliability and validity

To ensure the validity of the clinical data, all selected primary and secondary outcome measures (e.g., MoCA, WMS-IV, BBS, 6MWT) are highly standardized, widely validated, and culturally adapted for older Chinese populations with MCI. To maximize inter-rater reliability, all outcome assessors will undergo intensive pre-trial training and standardization sessions to ensure absolute consistency in test administration and scoring.

To minimize detection bias, the trial employs strict single-blinding; outcome assessors will remain completely blinded to participant group allocation across all time points (Baseline, Week 12, Week 24). Furthermore, intervention reliability (treatment fidelity) will be maintained through standardized operating procedures (SOPs). For the rTMS protocol, neuronavigation will be utilized to guarantee precise and consistent targeting of the left DLPFC across all sessions. For the DMI, adherence to the choreographic protocol and targeted exertion levels (Borg Scale 4-6) will be continuously monitored by supervising medical staff.

#### 2.6.2 Blinding integrity assessment

To rigorously evaluate the success of the single-blind design and ensure that treatment effects are not confounded by participant expectations, a formal assessment of blinding integrity will be conducted. At the conclusion of the 12-week intervention phase (T1), all participants will be asked to complete a standardized blinding questionnaire. Participants will be asked to guess their assigned intervention arm (e.g., “Active iTBS + Dance,” “Sham iTBS + Dance,” etc.) and to state the primary reason for their guess (e.g., perceived physical sensations, perceived cognitive improvement, or random guess) (Kolahi et al., 2009).

The blinding efficacy will be quantitatively evaluated using Bang’s Blinding Index (BBI), a validated statistical metric that ranges from -1 (complete opposite guessing) to 1 (complete unblinding), with 0 indicating perfect blinding (random guessing) (Bang et al., 2004). The BBI will be calculated separately for the active and sham iTBS conditions to determine if the sham coil successfully mimicked the somatic sensations of active stimulation without unmasking the participants (Berlim et al., 2013). If the BBI indicates significant unblinding, the participant’s guess will be included as a covariate in the secondary sensitivity analyses to adjust for potential expectancy biases.

#### 2.6.3 Qualitative trustworthiness

For the embedded qualitative interviews, methodological rigor will be established through the widely accepted framework of trustworthiness, comprising the four interrelated criteria of credibility, dependability, confirmability, and transferability.

Credibility (Internal Validity). To ensure that the analytical findings authentically reflect participants’ lived realities, member checking will be employed. Following transcription, interview transcripts will be returned to the respective participants to verify their accuracy and to confirm that participants’ experiences, perceived benefits, and reported challenges have been faithfully captured. Data triangulation will be further achieved by eliciting and contrasting perspectives from both the patients receiving the intervention and the medical staff delivering it, thereby strengthening the validity of the emergent themes.

Dependability (Reliability). To minimize individual interpretive bias and establish a robust coding framework, data analysis will be conducted independently by no fewer than two qualitative researchers. Each researcher will code the transcripts separately, and any discrepancies in thematic generation will be reconciled through consensus meetings. NVivo software will be used to support this process by maintaining a transparent and traceable audit trail throughout the coding procedure.

Confirmability (Objectivity). It is acknowledged that the researchers’ prior clinical experience and professional background may inadvertently shape both the interview process and subsequent data interpretation. To mitigate this risk, researchers will engage in active reflexivity by maintaining reflective journals throughout the data collection and analysis phases, enabling them to identify and bracket personal assumptions in order to preserve interpretive objectivity.

Transferability (External Validity). To allow future researchers and clinicians to evaluate the applicability of the qualitative findings to other contexts, rich and thick descriptions will be provided. By comprehensively detailing the clinical setting, the demographic characteristics of participants with MCI, and the specific nature of the combined rTMS and DMI interactions, readers will be sufficiently equipped to assess the relevance of this multimodal intervention to broader geriatric populations.

### 2.7 Sample size

A priori power analysis (G*Power 3.1) was conducted for a four-group repeated measures ANOVA (interaction effect). To detect a medium effect size (f = 0.25) with a power of 0.8 and an alpha level of 0.05, a total sample of approximately 128 participants (32 per group) is required. To account for potential participant dropouts, we have factored in an anticipated attrition rate of 20% over the 24-week study period (12-week intervention plus 12-week follow-up). This 20% estimate is intentionally conservative and rigorously justified by previous longitudinal literature involving older adults with cognitive impairment. Systematic reviews of intensive, multi-modal non-pharmacological interventions (including aerobic exercise and neuromodulation protocols) in geriatric populations consistently report attrition rates ranging from 15% to 21%, typically due to transportation barriers, intervention fatigue, or unrelated emergent health issues (Di Lorito et al., 2020). Therefore, to ensure the trial remains adequately powered throughout the follow-up phase, we aim to recruit a final total of 160 participants (40 per group).

### 2.8 Randomization-sequence generation and blinding

Participants will be randomly allocated to one of the four groups using a computer-generated randomization sequence. Allocation concealment will be ensured using sealed, opaque envelopes, which will be opened by a researcher not involved in assessment or intervention delivery. This is a single-blinded trial. Outcome assessors will be blinded to the group allocation of participants. Due to the nature of the interventions, participants and instructors cannot be blinded. However, participants will be instructed not to discuss their intervention with the assessors.

### 2.9 Statistical methods

#### 2.9.1 Quantitative statistics

All quantitative data analyses will be performed using standard statistical software (R, version 4.2). To ensure methodological rigor, the primary efficacy analysis will be conducted in accordance with the intention-to-treat (ITT) principle, encompassing all randomized participants regardless of adherence to the intervention protocols or study completion. A per-protocol (PP) analysis, restricted to participants who complete a pre-defined minimum number of intervention sessions, will additionally be performed as a sensitivity check to assess the robustness of the findings.

##### 2.9.1.1 Descriptive statistics and baseline comparability

Baseline demographic and clinical characteristics will be summarized across all four groups. Continuous variables will be presented as means with standard deviations (SD) or as medians with interquartile ranges (IQR), depending on the normality of the data distribution as assessed by the Shapiro-Wilk test. Categorical variables will be reported as frequencies and percentages. Baseline differences among the four parallel groups will be examined using one-way analysis of variance (ANOVA) for normally distributed continuous data, the Kruskal-Wallis test for non-parametric data, and the Chi-square or Fisher’s exact test for categorical data.

##### 2.9.1.2 Analysis of primary and secondary outcomes

To evaluate the individual and synergistic effects of the interventions across the three assessment time points (baseline, Week 12, and Week 24), linear mixed-effects models (LMMs) will be employed. LMMs are particularly well-suited to this trial design, as they accommodate repeated measures, account for intra-subject correlation, and appropriately handle missing data under the missing-at-random (MAR) assumption without necessitating listwise deletion. The outcome measures—including global cognitive function (MoCA), memory, executive function, EEG/ERP parameters, and physical performance metrics—will be modelled as dependent variables. Fixed effects will comprise Group (Combined Intervention, rTMS Only, DMI Only, Control), Time (T0, T1, T2), and the Group × Time interaction term, with the latter serving as the primary indicator of intervention efficacy over the study period. Participant identification will be specified as a random intercept to account for between-subject variability.

##### 2.9.1.3 Factorial analysis for synergistic effects

To directly address the study’s aim of elucidating synergistic mechanisms and to capitalize on the robust factorial design, a 2 × 2 factorial analysis will be nested within the models. The main effect of the rTMS factor (active vs. sham), the main effect of the DMI factor (active vs. sham), and the rTMS × DMI interaction effect on the primary outcome will be tested. A statistically significant positive interaction term will quantitatively support the hypothesis that the combined intervention produces benefits exceeding the additive effects of the individual modalities.

##### 2.9.1.4 Covariates and adjustments for multiplicity

The models will be adjusted for pre-specified, clinically relevant covariates, including age, gender, educational attainment, and baseline MoCA scores. Should baseline imbalances be detected across groups despite randomization, the affected variables will additionally be incorporated as covariates. For multiple pairwise comparisons across groups or time points, *post-hoc* analyses will be adjusted using either the False Discovery Rate (FDR) method or the Bonferroni correction to control the family-wise error rate.

##### 2.9.1.5 Handling of missing data

Although LMMs intrinsically accommodate missing data, multiple imputation (MI) via chained equations will be applied as a supplementary sensitivity analysis if the overall missing data rate exceeds 10% (in light of the anticipated 20% attrition rate), thereby corroborating the validity of the primary ITT findings. For all analyses, a two-sided p-value of less than 0.05 will be considered statistically significant.

#### 2.9.2 Qualitative statistics

Transcribed interview data will be analyzed using thematic analysis, following the six-phase approach outlined by Braun and Clarke (Braun and Clarke, 2006). Data will be coded independently by two researchers, and discrepancies will be resolved through discussion to enhance trustworthiness. NVivo software (Version 14.24.3) will be used to assist with data management and coding. Key themes related to acceptability, feasibility, and perceived impact will be identified and interpreted.

#### 2.9.3 Mixed-methods data integration

To maximize the explanatory power of this study, the integration of quantitative and qualitative data will occur at the interpretation phase using an embedded mixed-methods framework. Rather than analyzing the data streams in isolation, the qualitative findings will be systematically used to contextualize, explain, and expand upon the quantitative results. For example, if the quantitative linear mixed-effects models reveal that the Combined Intervention group achieves high clinical efficacy but suffers from unexpectedly low adherence or high attrition rates, the qualitative data will be crucial in identifying the underlying mechanisms—such as the perceived logistical or physical burden of attending both the iTBS and dance sessions. Conversely, if specific cognitive domains (e.g., executive function) show significant improvement, the interview transcripts will be analyzed to uncover the specific components of the DMI or social interactions that participants felt contributed most to those gains. This synergistic integration will provide a comprehensive understanding of not just *whether* the intervention is effective, but *how* and *why* it works, thereby yielding highly actionable insights for future real-world clinical implementation.

### 2.10 Outcome hierarchy and multiplicity adjustment

To address concerns about multiplicity, the trial outcomes are organized in a pre-specified hierarchy. The primary outcome is the change from baseline (T0) to post-intervention (T1) in MoCA total score. Three pre-specified key secondary outcomes, all analyzed as T0 to T1 changes paralleling the primary, are: (1) change in executive function, operationalized as the TMT-B/A ratio to control for motor speed, with TMT-B and TMT-A reported separately as supporting analyses; (2) change in P300, assessed by peak latency (ms) and peak amplitude (μV) at midline electrodes (Fz, Cz, Pz); and (3) change in BBS score. To control the family-wise Type I error rate across these three key secondary outcomes, the Holm-Bonferroni step-down procedure will be applied; effect estimates with 95% confidence intervals (unadjusted and adjusted) will be reported, with the adjusted p-values used for inference. T0 to T2 changes (durability) for all three key secondary outcomes, as well as all other pre-specified outcomes—including WMS-IV (memory), 6MWT (functional exercise capacity), SF-12 (quality of life), and rs-fMRI (DMN and frontoparietal connectivity)—are treated as supportive/exploratory and are interpreted with caution.

## 3 Discussion

### 3.1 Rationale for a multimodal approach

The primary objective of this study is to investigate the clinical efficacy of a combined repetitive transcranial magnetic stimulation (rTMS) and Chinese square dance intervention on cognitive function, neuroplasticity, and physical performance in older adults with mild cognitive impairment (MCI). A 2×2 factorial design is being used to disentangle the independent and combined contributions of these two modalities. The rationale is that iTBS-induced modulation of cortical excitability and DMI-induced upregulation of neurotrophic and cardiovascular pathways are hypothesized to converge on shared neurobiological mechanisms relevant to cognition in MCI (Cirillo et al., 2017). iTBS offers practical advantages (approximately 3-min sessions), and the combined protocol is hypothesized to capitalise on a potential priming effect, although this synergy remains to be tested empirically (Givralli et al., 2026).

### 3.2 Insights from previous studies on behavioral symptoms and engagement

Apathy is a clinically meaningful target in MCI, as reduced motivation may limit participation in lifestyle interventions. iTBS over the left DLPFC has been reported to improve both global cognition and neuropsychiatric symptoms in MCI (Chu et al., 2021; Han et al., 2026). We hypothesize that, if present, rTMS-induced improvements in motivation and drive may support higher adherence to subsequent DMI sessions; this mediation effect will be interpreted cautiously given the sample size.

### 3.3 Social engagement and cultural accessibility

The group-based, culturally familiar format of Chinese square dancing is hypothesized to address social isolation, a modifiable risk factor for cognitive decline (Read et al., 2020), by combining physical activity with structured social interaction. Observational data further suggest an association between long-term Chinese square dancing and preserved cognitive performance in older adults (Zhao et al., 2024); the present trial, however, is not designed to isolate the social contribution from the rTMS effect.

### 3.4 Outcome measures and clinical relevance

The selected outcomes capture cognitive, physical, and behavioral dimensions most relevant to MCI. MoCA provides a measure of global cognition; BBS and 6MWT capture motor and cardiovascular function relevant to independence and fall risk. Qualitative interviews will contextualize feasibility and acceptability from participant and staff perspectives.

### 3.5 The relationship between education level and intervention efficacy

Cognitive reserve, shaped by education, may modulate how older adults engage with combined cognitive-physical interventions. Because Chinese square dancing demands continuous attention, working memory, and spatial planning to synchronize complex movements with music, individuals with higher educational attainment may adapt more readily. We hypothesize that rTMS-induced modulation of cortical excitability may lower the cognitive threshold for learning these routines, potentially allowing adults with lower educational backgrounds to derive comparable benefits; this hypothesis will be tested in a pre-specified subgroup analysis.

### 3.6 Adverse events and safety in the older adult population

rTMS-associated adverse events in older adults are typically mild and transient (Rossi et al., 2021). The DMI protocol includes heart-rate monitoring and a seated option to limit fatigue and fall risk.

### 3.7 Ethics and dissemination

#### 3.7.1 Ethical approval and conduct

This study protocol has been reviewed and formally approved by the Research Ethics Committee, Ethics Committee of Handan Central Hospital (Approval Number: (2026) Ethics Review Paper No. (078)). All trial procedures, including both the quantitative interventions and the qualitative interviews, will be conducted in strict adherence to the ethical principles outlined in the Declaration of Helsinki and all relevant national regulations governing human subject’s research.

#### 3.7.2 Informed consent

Participation in this study is entirely voluntary. Prior to enrollment and any study-related procedures, all eligible participants—and their legally authorized representatives or family members, as appropriate—will receive comprehensive information regarding the study’s objectives, the nature of the interventions (rTMS and Chinese square dancing), potential risks, and expected benefits. Written informed consent will be obtained from all participants for the main randomized controlled trial. Furthermore, participants purposively selected for the embedded qualitative component will be required to provide an additional, specific written informed consent prior to participating in the interviews and being audio-recorded.

#### 3.7.3 Right to withdraw

Participants will be explicitly informed of their right to withdraw their consent and discontinue participation in either the main trial, the qualitative interview, or both, at any time. They will be assured that withdrawal can be done without providing a reason and will not result in any adverse consequences to their ongoing medical care, treatment, or relationship with Handan Central Hospital.

#### 3.7.4 Data confidentiality and security

Strict protocols will be implemented to safeguard participant privacy and data confidentiality. All collected clinical data, demographic information, and qualitative transcripts will be completely de-identified and assigned a unique, randomized study code. Original paper consent forms and physical assessment records will be stored in a locked filing cabinet within a restricted-access research office. All electronic data, including coded databases and audio recordings, will be encrypted and stored on secure, password-protected hospital servers. Access to any identifying information will be strictly limited to the principal investigator and the core research team. Following the completion of the study and publication of the results, all data will be securely retained for a minimum of five years before being permanently destroyed in accordance with institutional data governance policies.

#### 3.7.5 Safety monitoring and serious adverse event reporting protocol

An Adverse Event (AE) is defined as any untoward medical occurrence in a participant during the study, regardless of its causal relationship with the intervention. Expected, mild AEs (e.g., transient scalp discomfort from iTBS, mild delayed-onset muscle soreness from the DMI) will be managed conservatively by the supervising medical staff and documented in the participant’s case report form (CRF).

A Serious Adverse Event (SAE) is strictly defined as any event that results in death, is life-threatening, requires inpatient hospitalization, or results in persistent or significant disability/incapacity. In the event of an SAE, a rapid-response reporting protocol will be initiated. The supervising trial instructor must notify the Principal Investigator immediately (within 24 hours of awareness). The Principal Investigator is then legally and ethically mandated to formally submit a detailed SAE report to the Medical Ethics Committee of Handan Central Hospital within 48 hours. Furthermore, the trial steering committee will evaluate the SAE to determine if trial protocols require modification or suspension, and the event will be officially updated and disclosed on the Chinese Clinical Trial Registry in accordance with international trial transparency guidelines.

#### 3.7.6 Dissemination

The findings of this trial will be disseminated through multiple channels to maximize their scientific and clinical impact. The primary results, encompassing both the quantitative efficacy outcomes and the qualitative findings from the embedded interview component, will be submitted for publication in a peer-reviewed, open-access journal. Interim and final results will also be presented at relevant national and international academic conferences in the fields of neurology, cognitive rehabilitation, and geriatric medicine.

In accordance with international clinical trial transparency guidelines, the trial registry record (ChiCTR2600127655) will be updated with summary results within 12 months of study completion. A lay summary of the findings will be provided to all participants upon request. Authorship of all publications arising from this study will adhere to the criteria established by the International Committee of Medical Journal Editors (ICMJE). No professional medical writers will be employed without full disclosure in the acknowledgements section.

## 4 Conclusions

We hypothesize that the combined rTMS and DMI group will demonstrate superior and more durable improvements in global cognition and physical performance compared to the monotherapy or control groups. By providing a comprehensive, mixed-methods evaluation of this synergistic approach, this trial aims to inform future clinical guidelines and offer a feasible, culturally appropriate strategy for managing MCI in the aging population.

## Data Availability

Publicly available datasets were analyzed in this study. This data can be found here: This is a study protocol, the data is not available currently.

## Glossary

AD: Alzheimer’s disease
AE: Adverse Event
ANOVA: Analysis of Variance
BBI: Bang’s Blinding Index
BBS: Berg Balance Scale
BDNF: Brain-Derived Neurotrophic Factor
ChiCTR: Chinese Clinical Trial Registry
CONSORT: Consolidated Standards of Reporting Trials
COREQ: Consolidated Criteria for Reporting Qualitative Research
CRF: Case Report Form
DLPFC: Dorsolateral Prefrontal Cortex
DMN: Default Mode Network
DMI: Dance movement intervention
EEG: Electroencephalography
ERP: Event-Related Potential
FDR: False Discovery Rate
GDS-15: 15-item Geriatric Depression Scale
ICMJE: International Committee of Medical Journal Editors
IGF-1: Insulin-like Growth Factor 1
IQR: Interquartile Range
ITT: Intention-to-Treat
iTBS: Intermittent Theta Burst Stimulation
LMM: Linear Mixed-Effects Model
LTP: Long-Term Potentiation
MAR: Missing at Random
MCID: Minimal Clinically Important Difference
MCI: Mild cognitive impairment
MI: Multiple Imputation
MMSE: Mini-Mental State Examination
MoCA: Montreal Cognitive Assessment
NIA-AA: National Institute on Aging-Alzheimer’s Association
P300: P300 Event-Related Potential
RCT: Randomized Controlled Trial
rMT: Resting Motor Threshold
rs-fMRI: Resting-State Functional Magnetic Resonance Imaging
rTMS: Repetitive transcranial magnetic stimulation
SAE: Serious Adverse Event
SD: Standard Deviation
SF-12: 12-item Short Form Health Survey
SOP: Standard Operating Procedure
SPIRIT: Standard Protocol Items, Recommendations for Interventional Trials
TMT: Trail Making Test
6MWT: 6-Minute Walk Test
WMS-IV: Wechsler Memory Scale, Fourth Edition
